# Development of an adherence-enhancing intervention in topical treatment termed the topical treatment optimization program (TTOP)

**DOI:** 10.1007/s00403-014-1475-5

**Published:** 2014-06-04

**Authors:** Kristian Reich, Ulrich Mrowietz, Eleni Karakasili, Ina Zschocke

**Affiliations:** 1 Dermatologikum Hamburg, Stephansplatz 5, 20354 Hamburg, Germany; 2 Department of Dermatology, Psoriasis-Center, University Medical Center Schleswig-Holstein, Schittenhelmstraße. 7, 24105 Kiel, Germany; 3 SCIderm GmbH, Drehbahn 1-3, 20354 Hamburg, Germany

**Keywords:** Adherence, Intervention, Topical therapy, Psoriasis, TTOP

## Abstract

Psoriasis is a common, disabling, chronic, relapsing, inflammatory disorder of the skin with a worldwide prevalence of 2–3 % in which adherence to treatment is often poor. The majority of individuals have limited disease that is being treated with topical medication according to existing guidelines. Adherence rates are lower for topical compared with systemic treatment. Low medication adherence is a major problem for patients with chronic disorders as it results in suboptimal treatment outcomes, increased risk for development of concomitant diseases, inefficient use of health resources and considerable losses to society. However, to date no adherence-enhancing intervention has been developed for psoriasis patients under topical treatment. In this article, we report the development of the topical treatment optimization program (TTOP). The TTOP intervention aims to improve the information given to the patients and to result in an engaged patient–physician relationship. Application of the TTOP intervention in daily clinical practice may lead to a significant increase of adherence and the successful management of psoriasis and other chronic skin disorders.

**Electronic supplementary material:**

The online version of this article (doi:10.1007/s00403-014-1475-5) contains supplementary material, which is available to authorized users.

## Introduction

Poor adherence hinders the successful treatment of chronic conditions with efficacious available medication. Research during the past several decades showed that, depending upon the condition severity and the complexity of the prescribed regimen, as many as 40 % of patients do not adhere to the agreed treatment recommendations [50]. In its 2003 report, the WHO recognized that “increasing the effectiveness of adherence interventions may have a far greater impact on the health of the population than any improvement in specific medical treatments” [67].

Adherence among patients with chronic conditions is disappointingly low, dropping most dramatically after the first 6 months of treatment [60]. It has been suggested that, regardless of their condition, treatment or age, only 50 % of individuals with chronic conditions adhere to their treatment [64]; [28, 67, 68]. Low medication adherence leads to an inefficient use of health care resources and therefore creates a considerable financial loss to society. Furthermore, low adherence can result in a misleading clinical picture in a patient’s treatment: if physicians erroneously assume that their patients have taken prescribed medication, they may make inappropriate medication and/or dosage changes, which in turn could result in suboptimal health outcomes [50]. Importantly, it has also been shown that poor adherence in psoriasis also increases the risk for the development of concomitant diseases, such as depression, inflammation-related coronary heart disease (CHD)/stroke, diabetes and cancer [27, 35, 58].

Psoriasis is a common skin disease with a prevalence of ~2–3 % thereby affecting around 25 million people in North America and Europe [48]. Importantly, more than 90 % of patients have a chronic course and therefore require continuous control of disease activity [56]. There is a high disease burden associated with psoriasis, including various psychosocial impairments and a significant reduction of the patient’s quality of life [2, 24, 37, 43, 49, 54]. Furthermore, the financial burden on society associated with psoriasis is substantial [70, 72]. Therefore, successful management of this chronic condition is crucial.

Since the majority of psoriasis patients have mild or moderate disease, topical corticosteroids, which reduce inflammation and decrease the rate of skin cell growth, are the most commonly prescribed topical treatment for psoriasis [45].

Given the specific aspects of a topical treatment such as the cosmetic and galenic properties as well as the time required for and the convenience of its application it is not surprising that in general topical treatment medication adherence for dermatological conditions is poor, with more than 30 % of the prescriptions to first time outpatients never being redeemed [75]. It has been reported that the correct number of medication applications per day declined over time (only 77 % after 2 weeks and only 50 % after 8 weeks) [11, 67, 75]. However, for patients with psoriasis, the reported primary adherence (redemption of initial prescription) is even lower reaching only 65 % while secondary adherence (correct use of medication) is even lower with 39–73 % of the patients not using the medication as prescribed [3, 6, 8, 22, 64, 75, 81]. In a study of almost 1,300 psoriasis patients 73 % of the patients reported not adhering to their treatment with the most frequently reported reasons for this being low efficacy (27 %), poor cosmetic characteristics (29 %) and time needed for the application (26 %) [25]. In another study with more than 800 psoriasis patients, 72 % of who had used topical steroids in the previous 6 months reported that only 51 % of the patients used their topical medication as prescribed [79]. All these studies have highlighted the high prevalence of non-adherence among psoriasis patients treated with a topical medication. As a consequence, the current view of physicians on topical treatment in psoriasis is unfortunately less favorable than it should be, if the clinical efficacy of these treatments is considered. Therefore, the prescription behavior of physicians is influenced towards other, oral treatments, even when prescription guidelines direct differently [54].

In spite of the large number of studies that have identified the importance of medication non-adherence, there are relatively few studies reporting and designing effective strategies to improve adherence. Given that the barriers to medication adherence are complex and varied, solutions to improve adherence must be multi-factorial [9]. A recent review of the literature which was done for the Cochrane database, reported that as far as the treatment of chronic conditions is concerned, adherence-enhancing interventions—none of which was designed for psoriasis—were not significantly effective and therefore the authors highlighted the need for designing comprehensive and innovative interventions which would improve medication adherence [28]. Furthermore, it is of high importance to design an adherence-enhancing intervention that on the one hand can be used at the everyday clinical routine and on the other hand can increase a patient’s adherence to the extent that the efficacy of the prescribed medication is observed in the general population.

Here we report the development of an adherence-enhancing intervention in topical treatment, termed topical treatment optimization program or shortly TTOP. This tool is created to address patients’ non-adherence in topical treatment and the resulting underperformance of such treatments in controlling psoriatic disease.

## Methods

### Literature search

A systematic literature search of the Medline database was conducted in December of 2010 and January of 2011. The aim was to generally identify important factors that influence a patient’s adherence to the prescribed treatment as well as vital characteristics that should be incorporated in an adherence-enhancing intervention. The search strategy included terms for adherence (adherence, non-adherence, compliance, non-compliance), treatment (medication, drug, treatment, regimen), chronic conditions (chronic conditions, psoriasis) and clinical trial design (clinical trial, intervention, outcome, randomized) [(((((((((adherence) OR non-adherence) OR compliance) OR non-compliance)) AND ((((medication) OR drug) OR treatment) OR regimen))) AND ((((clinical trial) OR intervention) OR outcome) OR randomized))) AND ((chronic conditions) OR psoriasis)], which provided 466 hits.

### Patient qualitative focus group interviews

To assess all relevant aspects, needs and views of psoriasis patients, focus group interviews were conducted in February and March of 2011, one with a national [4 members (3 men and 1 woman) of the German Patient’s Psoriasis Association—Deutscher Psoriasis Bund e. V.] and another with an international [6 members (2 from Germany, 1 each from Spain, Denmark, Sweden, and The Netherlands) of the European Federation of Psoriasis Patient Organization (EUROPSO)] patient advisory board. Patients participating in the interviews were selected according to their experience with topical treatment and in general with managing psoriasis and not according to their psoriasis condition. The aim was to have a representative population as the TTOP intervention could be applied for all patients with topical treatments. Guided by an interviewer, participants were asked to give their feedback on the following questions: “What are the main characteristics of a typical visit of psoriasis patients at their dermatologist?”, “What actions could be done in order to improve the quality of the consultations and the treatment that the patients receive at the beginning or during the course of the treatment?”, “What information should be provided during a dermatologist-patient consultation?”, “What information should be provided during a nurse–patient consultation?” and “What are important points/aspects on disease and topical medication to be provided in an information brochure for patients?”.

### Expert qualitative focus group

A total of 11 experts from the fields of dermatology (7), psychology (1), health economic (1) and clinical research (2) were invited to participate in an expert panel meeting in April 2011. The corresponding experts reviewed each intervention element, either identified via the literature search or during the patient interviews, and its relevance and suitability were evaluated for inclusion or exclusion from the newly developed adherence-enhancing intervention TTOP.

The outcomes of the literature search, patient qualitative focus group interviews and expert qualitative focus group are presented in the results section.

## Results

### Literature search

Citation titles, index terms, and abstracts were screened to identify potentially relevant articles, which were subsequently retrieved for full-text review. Original articles were selected if they reported the development and application of at least one adherence-enhancing intervention in the treatment of chronic medical conditions [5, 13, 17, 19, 29, 32, 36, 38, 41, 44, 53, 57, 59, 61, 66, 69, 71, 74]. Furthermore, the selected studies were required to report one measure for assessing medication adherence and/or assessment of one clinical outcome for showing effectiveness of the intervention. Additionally, review articles that summarized the current status of research on adherence-enhancing interventions in chronic conditions were also selected and corresponding references therein were retrieved for full-text review [1, 26, 34, 40, 46, 55, 73, 80]. Furthermore, 14 publications, which were not identified via the literature search but which were known to the authors as being relevant, were included in the pool of the literature retrieved for full-text review [10, 15, 20, 21, 23, 31, 39, 47, 52, 62, 63, 65, 76, 77]. Given that none of the already published adherence-enhancing interventions were specific for psoriasis it was difficult to identify factors specific to psoriasis or its treatment.

In summary, the following areas were identified as being important to be incorporated in an adherence-enhancing intervention: simplification of regimen, understanding of treatment instructions, comorbidity and overall health status, patient-reported outcomes, quality of life, treatment satisfaction, quality of patient–doctor relationship, patient education, information regarding the condition and its treatment, self-reported adherence, objective assessment of adherence and involvement of patient in treatment decision-making process.

Subsequently, intervention elements were created that were included within the aforementioned areas. These areas were presented for discussions with both the patient and the expert qualitative focus groups.

### Patient qualitative focus group interviews

Discussions with the national patient board resulted in the proposal to create a checklist specifying and allocating necessary tasks (either for the treating physician or other health care professionals), which should be followed during the patients’ consultations both at the start of the treatment as well as during the course of the treatment. Based on the patients’ proposal these checklists should instruct the health care provider: (1) to provide information regarding the disease, potential treatments and potential further treatments in case of disease exacerbation, (2) to explain actual chosen therapy and motivate the patient to ask questions and communicate expectations regarding the therapy, (3) to pose questions on skin condition and other areas of the patient’s life and (4) to hand out information material. Second proposal, originating from the patient interviews was to install a function in the practices/hospitals serving as contact point for the patients between the regular visits.

The proposals of the patients from the national board were taken into account so that at the time of the international patient board, a preliminary checklist for physicians (or other health care professional) and a preliminary patient questionnaire concerning the topical treatment were created. Both preliminary tools, which additionally included items resulting from the literature search, were discussed during the interview with the international patient board. Regarding the checklist, new aspects which were identified during the interview were included in the checklists which were later placed for discussion during the expert qualitative focus group. Regarding the questionnaire, all points collected during the meeting were covered by the current items of the item pool. Later analysis of the results of these interviews revealed that all aspects reported by the patients were already included in the element pool that had been created from the literature search. In general, the patients from both boards expressed their wish for a holistic approach from health care providers, with time to talk and learn about the disease and the therapy, both at the start of a treatment as during the course of the therapy/disease.

### Expert qualitative focus group

All items gathered by either the literature search or by the qualitative patient focus group interviews were provided to the expert focus group and discussed. Regarding the task and allocation guidelines the experts discussed and shorted out all of the different items and finalized the content as well as structure of two guidelines; one intended to be used by the treating physician and one to be used by the nurse. Regarding, the patient questionnaire once again all items were discussed and the experts decided which of these should be included in the final version of the questionnaire. Importantly, it was proposed to rephrase all items as statements rather than questions.

Furthermore, the experts discussed which other elements should be included in the developed intervention. It was agreed to provide the patients with information material in the form of a patient card containing information on the treatment and its application so that patients can remember easily how to apply their treatment. Finally, as further supporting information material the experts proposed to create an information brochure regarding psoriasis and its management. The content of all abovementioned documents is described in more detail below (section “Patient education”).

Resulting from the literature search, the interviews with both patient boards and the expert advisory board meeting, the TTOP has been developed with the aim to firstly qualitatively and quantitatively increase the information provided to the patients regarding their condition and its available treatment and secondly to improve the patient–doctor relationship to the extent that the communication between the two parties is optimal and does not hinder the patient’s adherence.

### The TTOP intervention and its elements

#### Patient education

The first key element of the developed TTOP intervention involves educating the patients regarding their condition and its treatment.

According to TTOP, patients receive extensive information regarding psoriasis and the treatment that is provided to them during one-to-one consultations with the treating physician as well as a nurse or other responsible members of the staff. For that purpose, standardized communication guidance in form of checklists, one for treating physicians (Online Resource 1) and one for nurses (Online Resource 2), was compiled resulting from the patient boards and expert advisory boards. At the beginning of their treatment patients are being educated by the physician regarding psoriasis’ pathophysiology (i.e., immune driven disease), regarding its chronicity (i.e., life-long, chronic), its comorbidity and various other facts associated with their condition (i.e, non-infectious, non-malignant). Furthermore, the physician explains to the patients how the psoriasis severity grade is being assessed according to the clinical manifestation of their condition and the severity of symptoms that each patient exhibits.

Subsequently, the physician discusses with the TTOP intervention patients the available treatments for psoriasis (i.e., topical, systemic and photo treatment) and the characteristics of each treatment are compared against each other. The physician then informs the patients that according to their condition severity, prescription of topical treatment is the ideal choice for them. In this way, the patients get involved by participating in the treatment decision-making process. Following this information, the physician introduces to the patient the study medication regarding its ingredients, considerations that need to be made before, during and after its application and regarding realistic treatment expectations. Patients are encouraged to consult the package information leaflet which accompanies the medication and are trained by the nurse how to correctly apply the medication to their affected areas. Additionally, within the TTOP intervention patients are also provided with a pocket card that describes the regimen and mode of application of their topical treatment. Furthermore, on the 7th and 21st day after treatment start patients are contacted—either by phone or email—by the nurse to be reminded to apply their topical medication daily. Importantly, to actively involve the patients in their treatment and to get their views and opinions regarding their treatment throughout its duration two novel questionnaires have been developed which aim to enable the early identification of potential non-adherent behaviors and adherence-enhancing factors and to assess the patient’s satisfaction with the current topical treatment: the Topical Treatment Adherence Questionnaire (TTAQ) and the Patient Preference Questionnaire (PPQ). The TTAQ, containing 59 items, assesses the following areas of the patient’s evaluation of topical therapy: “Patient’s benefit from treatment”, “Knowledge, communication and relationship with the physician” and “Patient satisfaction with treatment”. The PPQ, containing 10 items, was developed to collect data on the patients’ preference to the current treatment in comparison to their previous treatments. For both questionnaires, all items are scaled in a 4-point Likert format (0 = strongly disagree, 1 = disagree, 2 = agree and 3 = strongly agree), with a supplementary option to tick “Does not apply to me”. The development of TTAQ and PPQ was based on a systematic literature review, patient focus interviews and expert focus groups and is described in detail in a separate publication [82]. Finally, written information material is provided to the TTOP intervention patients in form of a brochure. Importantly, the brochure’s text is formatted in Questions & Answers aiming to increase its comprehensibility to the patients. The “TTOP brochure” contains short summaries of important information regarding psoriasis: “What is psoriasis?”, “What causes psoriasis?”, “Is there a cure for psoriasis?”, “How does clothing affect psoriasis?”, “What can trigger psoriasis?”, “What health complications are associated with psoriasis?”, “Is psoriasis linked to other diseases?”, “Does having psoriasis affect lifestyle or quality of life?”, “Why does psoriasis itch?”, “Is psoriasis contagious?”, as well as information regarding the treatment and types of treatment of psoriasis, and lifestyle including physical activity. Through all these intervention elements it is ensured that the patient understands thoroughly the treatment instructions, has realistic treatment expectations, has no anxieties over the complexity of the regimen and is reminded how and when to apply the prescribed medication.

#### Patient–health care provider relationship

The second key element of the TTOP intervention aims at improving the relationship and communication between the patients and their health care provider; both their treating physician and the nurse.

To that end, both the physician and the nurse are applying a number of approaches to improve their relationship with the patients treated according to the TTOP intervention. For this purpose, we have developed a guide (“A General Patient Encounter Guide”), which has been adapted from E.E. Uhlenhake et al. 2010 [78], stating several general considerations that need to be made during the encounter of the health- care providers with their patients (Online Resource 3). The physician and nurse are among others instructed to greet politely the patients at the beginning and the end of the visit, to lean forward towards the patient showing that they are engaging in the conversation with them, to provide enough time for the consultation, to ask open-ended questions (e.g., “How are you today?”, “Is there anything else?”), to not interrupt the patients, to ensure that the patients’ needs are acknowledged, to be open, honest and responding regarding any aspect of the treatment and to ask them how is their life affected by their condition and offer sympathy. Furthermore, as mentioned above according to TTOP the patients are informed to a high extent regarding their condition, the prescribed treatment and are encouraged to actively participate during their consultation. All these approaches aim at having the patients feel that their physician/nurse takes enough time to care, listen and respond to them thus establishing a personal relationship with them which is based on collaboration, trust, sympathy and understanding. Importantly, in order to feel supported by their physician, TTOP intervention patients are provided with the possibility to e-mail or telephone their health care provider in between visits (helpdesk), if specific questions/problems arise that the patient wishes to clarify and discuss. Finally, at each consultation the importance of adhering to the treatment is reminded to the patients and their feedback is being asked regarding potential challenges that they have encountered during their treatment.

## Discussion

No single intervention strategy could improve adherence of all patients. Discussions with both the German as well as the International patient boards indicated that a number of different factors are considered by different patients as being important for adhering to the prescribed treatment. Several studies have shown that by just increasing the information given to the patients or by solely improving the patient–physician communication or even by simplifying the prescribed treatment will not result in increasing the adherence for all patients [50]. Moreover, several studies have attempted to describe, summarize and categorize factors and predictors of non-adherence as well as medication adherence-enhancing factors [3, 7, 9, 12, 16, 30, 33, 50, 51, 60, 64]. According to these studies, adherence is a multidimensional phenomenon determined by the interplay of a large number of factors which mainly fall into the following three categories: therapy-related factors (e.g., complexity of the medical regimen, interference of treatment with daily routine and duration of treatment), patient-related factors (e.g., severity of symptoms, level of disability, existence of comorbidity and misunderstanding of treatment instructions) and health care-related factors (e.g., quality of the patient–health care provider relationship, duration of consultations and capacity of the system to educate patients and provide follow-up). Therefore, the TTOP intervention was designed to target a multitude of these factors from all identified areas in order for the highest possible increase in medication adherence to be achieved.

Importantly, it is recognized that adherence and the act of following a treatment (e.g., ingesting a pill or applying a topical medication) is a certain a behavior. Therefore, theories of behavior change should be applied and taken into account upon designing an adherence-enhancing intervention [16]. Various elements from change behavior theories were addressed when developing the TTOP intervention. Since these theories share the central assumption that people are capable of forethought, planning, and rational decision making and that people experience their decision making and self-regulation as part of a dynamic social learning process [7] in the TTOP intervention patients are provided with an increased amount of information that would allow them to carefully think and decide that following their prescribed treatment and hence being adherent is a necessary behavior and is a decision originating from them.

The first characteristic of the developed intervention is to educate the patients regarding their condition and its treatment. Adjunctive treatment in the form of patient education is now widely advocated in chronic disease management and has been shown to be a simple but effective means of supplementing conventional treatment and contributing to satisfaction with care as well as treatment adherence [64]. By increasing the amount of information that they receive, patients feel that they are actively participating in improving their condition. Studies have shown that adherence is significantly enhanced by patients’ involvement and participation in their care. Based on the self-efficacy theory, where it is assumed that perceived self-efficacy is about what individuals believe they can do with the skills they have or what they can learn in various circumstances and situations [7], it has been suggested that the more empowered patients feel, the more likely they are to be motivated to manage their disease and adhere to their medications [9, 16]. Importantly, studies have shown that a major contributor to non-adherence is patients’ forgetfulness to take (or how to take) their prescribed medication [50]. Therefore, TTOP intervention patients are being reminded (either from their pocket cards or by directly contacting them) to adhere to their treatment.

The second characteristic of the TTOP intervention is improving the relationship of the patients with their health care provider. Several studies have reported that patients who report a communicative, understanding and encouraging relationship with their physician tend to be more motivated to adhere to their treatment [50]. The substantially improved adherence of these patients highlights the important role of physicians in increasing their patients’ adherence [9]. Effective communication is of course the key to a good physician–patient relationship. It is proposed that a patient-centered approach that consists of participation, engagement, encouragement, collaboration, negotiation, and sometimes compromise results in patients feeling responsible to adhere to their treatment and thus in optimal treatment outcomes [9, 50].

To potentially show the importance of an optimized contact between the patient and health care professional in increasing topical treatment adherence, the TTOP is currently being compared to standardized minimal regular care in an international phase IV study with ~2,000 patients. Assessing the effectiveness of the TTOP intervention should be multi-factorial. Initially, a very important aspect to be considered should be the standardization of the medication as it has been shown that one important factor leading to poor adherence is the frustration of the patient with messy and inconvenient formulations of topical therapies, leading from poor use of medication to not using the medication and/or not collecting prescriptions [4, 75]. The prescribed regimen has also been shown to significantly affect adherence with once-daily regimens resulting in 84 % adherence while for thrice-daily regimens reported adherence is only 59 % [50]. Therefore, it is of high importance that patients treated according to the TTOP intervention as well as patients receiving the standardized minimal regular care should be treated with a highly efficacious topical medication with a simplified regimen.

Furthermore, to assess the effectiveness of the TTOP intervention, it is imperative that the patient’s adherence as well as the medication’s efficacy is assessed. Adherence can be assessed in a variety of ways including pill counts, self-reports or patient diaries, physician reports, electronic monitoring of medication use, biochemical assays and biologic markers, medical record/chart and electronic pharmacy records [50, 51]. Ideally, adherence should be assessed in a two-way approach, both objectively (e.g., weight of used topical medication) as well as subjectively (e.g., self-reported patient adherence). The aim would be to assess if adherence rates of patients treated according to the TTOP intervention are higher than the ones of patients receiving the standardized minimal regular care. Furthermore, the improvement in their condition severity should be assessed. In addition to the commonly used clinical assessment methods (e.g., PGA, BSA) the patients should also be asked to self-assess their condition severity. To that end, we have developed a novel method that can be used for assessing psoriasis severity by the patients. This new tool is termed Patient’s Global Assessment or short PsGA and it is the patient equivalent of PGA (Table 1). Importantly, patients’ views and opinions should be regularly sought out by use of the newly developed patient questionnaires TTAQ and PPQ. Since there are the significant differences between the TTOP intervention and the standardized minimal regular care and to assure that patients will receive differential treatment, communication guidelines in form of checklists have been created. These guidelines define the structure and the sequence of the tasks that should be performed during each patient’s visit and according to the treatment that this patient is receiving (TTOP intervention or standardized minimal regular care). Moreover, it is also defined who would be responsible for performing which task (e.g., the nurse is responsible to train the patient how to correctly apply the medication). For the TTOP intervention, these guidelines shall also assist in the building of a more personalized relationship with the patients over time and provide ample opportunity for the health care professional to take the patient-desired holistic approach: helping the patient to learn about his disease and the treatment offered. Since the TTOP intervention is an adherence-enhancing intervention which depends on the increased amount of information and communication between physician and patient it is evident that consultation of patients according to TTOP has to be more time consuming than that of standardized regular care. To be able to assess how time consuming the implementation of the TTOP intervention into the daily routine practice would be, the study personnel at all study sites should be asked to document the time required for the consultation of each patient. Finally, to be able to assess which elements have to be included or not during the intervention’s implementation into daily practice, patients should also be asked to rank the different elements of the TTOP (helpdesk, patient brochure, one-to-one conversations with study nurse, one-to-one conversations with dermatologist, reminders sent to patient) using a 5-point Likert scale.

**Table 1:** Patient’s Global Assessment (PsGA)

Score	Category	Description
0	Clear	The skin looks normal with no signs of lesions
1	Almost clear	There are only faint lesions with some slight redness, barely elevated and a few scales
2	Mild	The skin lesions have a light red color, may be slightly elevated and a bit scaly
3	Mild to moderate	The skin lesions have a reddish color, are a bit elevated and somewhat scaly
4	Moderate	The skin lesions are clearly red, elevated and scaly
5	Moderate to severe	The skin lesions are really red, thick and with a lot of scales
6	Severe	The skin lesions are very red, really thick and with heavy scaling

Development of the patient’s global assessment (PsGA) tool was based on the commonly used clinical assessment physician global assessment tool [42] to be used for assessment of psoriasis’ condition severity by the patient

Given that when patients are more satisfied with their medical visits, they tend to experience better recall of information that they receive [18] the aim of the TTOP intervention is just that; having the patients leave the doctor’s office feeling satisfied with the time which was provided to them, the information that they received and the communication with their health care provider. As a consequence, the patients are more likely to adhere to their treatment achieving thus optimal treatment outcomes [14, 64].

The results of the ongoing study assessing the effectiveness of TTOP are eagerly awaited and will be published after termination of the study. In addition, it will also be interesting to determine what could/must be transferred into the daily routine clinical practice and how practical or time consuming such a transfer might be. In the long run, we aim to be able to have the key elements of the TTOP program being applied in the daily clinical routine leading hence to a significant increase of adherence and improvement of the severity grade of their chronic condition for the patients.

## Electronic supplementary material

Below is the link to the electronic supplementary material.

Supplementary material 1 (PDF 189 kb)

Supplementary material 2 (PDF 179 kb)

Supplementary material 3 (PDF 69 kb)
