# Supplementary material for: Development of an adherence-enhancing intervention in topical treatment termed the topical treatment optimization program (TTOP)
Source: Arch Dermatol Res. 2014 Jun 4;306(7):667–76. doi: 10.1007/s00403-014-1475-5 (PMC4139584; doi:10.1007/s00403-014-1475-5)
Supplement: Supplementary file 1 — Supplementary material 1 (PDF 189 kb) [file 403_2014_1475_MOESM1_ESM.pdf]

Development of an adherence enhancing intervention in topical treatment termed the Topical Treatment Optimization Program (TTOP)

Archives of Dermatological Research

Kristian Reich[a], Ulrich Mrowietz[b], Eleni Karakasili[c], Ina Zschocke[c*]

[a]Dermatologikum Hamburg, Stephansplatz 5, 20354, Hamburg, Germany

[b]Psoriasis-Center, Department of Dermatology, University Medical Center Schleswig-Holstein, Schittenhelmstraße. 7, 24105, Kiel, Germany

[c]SCIderm GmbH, Drehbahn 1-3, 20354, Hamburg, Germany

*Corresponding Author:
Ina Zschocke
SCIdem GmbH
Drehbahn 1-3
20354 Hamburg
Germany
Phone: +49-040-55 44 010
Fax: +49-40-55 44 01 291
Email: ina.zschocke@sciderm.com

Online Resource 1 Standardized communication guidance for physicians

At treatment start

**One to one with the patient**
**Psoriasis as a chronic disease: facts and implications**
Key phrases:<br>Evaluate patient's knowledge of psoriasis<br>• i.e. life-long, chronic, auto-immune driven, non infectious, non-malignant, increased risk for concomitant diseases, impact of unhealthy life-style<br>If appropriate, repeat/affirm or convey and explain this knowledge.<br>• Patient will be trained to perform the self-assessment of psoriasis severity (PsGA).<br>• Patient will meet with nurse.<br>• The nurse is the patient's personal contact during visits but also in between.<br>• The nurse will also hand-out/return medication, hand-out questionnaires and will schedule subsequent visits.

**Psoriasis disease severity**
Key phrases:<br>• Psoriasis affects people differently.<br>• Physicians grade severity of psoriasis based on rather rough criteria.<br>• Severity in 3 grades: mild, moderate or severe<br>• Severity characterized by: body surface affected, plaque area and severity of plaque<br>• Plaque severity characterized by: number of lesions, redness, elevation and scaling of skin<br><br>Inform patient about his severity grade.

**Introduction of topical treatments**
Key phrases:<br>• Current status of patient's skin especially suited for topical therapy<br>• Topical compared to systemic treatments: local effect eliminates risks for side effects associated with oral/intravenous therapy (tablets/drip).<br>• Topical compared to UV therapy: no increased risks for malignancies<br>• Background information on different formulations of topical treatments:<br>1. creams are mainly used for treatments of wet skin diseases.<br>2. ointments are mainly used for treatment of dry, scaling skin disease.<br>3. lotions are mainly used for treatments of 'itchy' skin diseases.<br>4. non-alcoholic gels can be applied for treatment of dry, scaling skin, similar to ointments.<br>• Explain selected topical treatment in detail.<br>• Explain treated regimen.<br>• Encourage patient to also read the package information leaflet that comes with his medication.<br>• Patient should inform you or nurse as soon as possible if he experiences any adverse events.<br>• Explain advantages of selected medication.<br>• Patient should be informed when to expect first visual effects.

**Referral of patient to nurse**
Key phrases:<br>• Lot of information today<br>• Any immediate questions before seeing the nurse?<br>• Encourage patient to write down questions and bring them to subsequent visit.<br>• Encourage patient to contact nurse/helpdesk if he has any immediate questions in between visits (specific telephone number and/or e-mail address available).<br>• Refer patient to nurse who is specialized in assisting psoriasis patients.<br>• Schedule next visit

Subsequent visits - summary

<table>
<tr><td><u>Welcome</u></td></tr>
<tr><td><u>Key phrases</u>:
• Well-being patient since last visit
• Any side effects / adverse events (when in a clinical trial)?
• Any change in medication?
• Any days away from work/studies due to psoriasis?
• Questions with regard to disease?
• Questions with regard to medication?
• Questions with regard to information discussed so far?
• Discussion with regard to life style factors which may influence psoriasis and general health
• Today doctor patient visit focus on PGA and BSA evaluations to check current status of psoriasis as well as discussion about impact of life style on psoriasis.</td></tr>
</table>

<table>
<tr><td><u>Information about impact of life-style on psoriasis, health risks (development of psoriasis-linked diseases) and overall quality of life</u></td></tr>
<tr><td><u>Key phrases:</u>
a) Part of successful disease management is the acceptance that psoriasis is a chronic and life-long disease.
b) A life style that is as healthy as possible/manageable will considerably reduce the risk for increased psoriasis flare-up periods and the beginning or worsening of psoriasis-linked diseases.
c) Lack of knowledge about the disease psoriasis by both the patient and his social circle (family, work, neighbours, school, etc) contributes to stress and social isolation.
d) Risk factors that are known to have a negative effect on psoriasis include: smoking, overweight, lack of exercise, alcohol overconsumption.

<u>Hand out a special TTOP Patient Information Brochure; key phrases:</u>
a) Contains a lot of information about psoriasis
b) Upon patient’s wish, this information can be discussed during coming visits.</td></tr>
</table>

<table>
<tr><td><u>Information about the impact of life-style on psoriasis, health risks and overall quality of life</u></td></tr>
<tr><td>’During your last visit we shortly touched upon the importance of a healthy life style as part of the successful disease management of psoriasis. This visit we could, if you so wish talk a little more about this topic.’

<u>Helpful key phrases, if discussion on life-style is continued:</u>
• Important to realize/know that psoriasis is a chronic and life-long disease.
• Psoriasis is not a contagious disease (not a virus / germ / fungus).
• Psoriasis is a quite common disease: 2-3% of the population in Europe has psoriasis, which means almost 2 million people.
• A healthy life style that is manageable for patients, reduces the risks for (increased) psoriasis flare-up periods and getting or worsening of other, concurrent diseases can be reduced.
• Psoriasis patients have an increased risk for other concurrent diseases like (psoriatic) arthritis, ‘sugar’ (diabetes) and heart diseases.
• Risk factors for concurrent diseases include stress, smoking, alcohol abuse, overweight, lack of exercise.

<u>Please select appropriate example below for further discussion, if applicable:</u>
<u>- Impact smoking on psoriasis</u>:
Smoking generates so-called reactive oxygen species in the body that cause damage and inflammation far beyond the lungs; the body, in the long run, cannot readily repair this damage. Simultaneously, the psoriasis is driven not only by the overproduction of new skin cells, but also by inflammation. The smoking-induced inflammatory reactions add on top of this psoriasis-induced inflammation and this of course worsens the general health and psoriasis status.

<u>- Impact overweight on psoriasis</u>:</td></tr>
</table>

Overweight is also an important risk factor in psoriasis. It especially increases the risks for heart disease and diabetes. A healthy diet (avoiding fast food and fried foods) and regular exercise - walking, swimming, cycling, nothing extreme - will help reducing this risk.

- Impact lack of knowledge on psoriasis:
The lack of understanding of the disease psoriasis in society (family, work, neighbours, school, etc) has high impact on the patient's quality of life.

Ranking of TTOP elements

Ask patient to rank the subjective importance of the different TTOP elements from the patient's perspective:

Rank 1 to 5 in order of importance (1 = most important; 5 = least important):

☐ Helpdesk (e-mail and telephone)
☐ Information on paper handed out so far (TTOP Patient Information Brochure)
☐ One to one conversations with nurse
☐ One to one conversations with dermatologist
☐ Reminders sent to patient

Referral of patient to nurse

- Inform patient about end of current treatment
- However, psoriasis is life-long current disease that will continue to flare up and may get worse over time.
- Encourage patient to visit you (or another dermatologist, if appropriate) on a regular basis
- Prescribe medication as necessary.
- Ask patient if there are any questions left unanswered that patient might want to ask today.
- Thank patient for participation in study.
- Refer patient to nurse.
