# Supplementary material for: Development of an adherence-enhancing intervention in topical treatment termed the topical treatment optimization program (TTOP)
Source: Arch Dermatol Res. 2014 Jun 4;306(7):667–76. doi: 10.1007/s00403-014-1475-5 (PMC4139584; doi:10.1007/s00403-014-1475-5)
Supplement: Supplementary file 2 — Supplementary material 2 (PDF 179 kb) [file 403_2014_1475_MOESM2_ESM.pdf]

Development of an adherence enhancing intervention in topical treatment termed the Topical Treatment Optimization Program (TTOP)

Archives of Dermatological Research

Kristian Reich[a], Ulrich Mrowietz[b], Eleni Karakasili[c], Ina Zschocke[c*]

[a]Dermatologikum Hamburg, Stephansplatz 5, 20354, Hamburg, Germany

[b]Psoriasis-Center, Department of Dermatology, University Medical Center Schleswig-Holstein, Schittenhelmstraße. 7, 24105, Kiel, Germany

[c]SCIderm GmbH, Drehbahn 1-3, 20354, Hamburg, Germany

*Corresponding Author:
Ina Zschocke
SCIdem GmbH
Drehbahn 1-3
20354 Hamburg
Germany
Phone: +49-040-55 44 010
Fax: +49-40-55 44 01 291
Email: ina.zschocke@sciderm.com

Online Resource 2 Standardized communication guidance for nurses

At treatment start

**Introduce yourself to patient and explain your role**
Key phrases:<br>• Patient's personal contact and support during and in between consultations<br>• Available for help<br>• Training the correct application of the medication and develop a treatment plan with patient<br>• Responsibilities study nurse:<br>Hand-out/return questionnaires<br>Hand-out/return medication<br>Scheduling of visits<br>• I can be reached during working hours at special phone number.<br>• I can be reached over e-mail.<br>• My contact details are written on the Patient Card.<br><br>Hand-out Patient Card with contact details.

**Localisation of psoriasis**
'Please name/show me all psoriasis affected skin areas, so that I can get an idea concerning<br>(a) which plaque would be best for training the application of the medication with you,<br>(b) the time needed on a daily basis in the coming weeks and<br>(c) the help you might need at home for the topical treatment, if applicable.'

**Training application of medication and its special aspects**
• Train application of topical treatment with patient; give feedback and optimise technique.<br>• If necessary, explain your reasons for your wearing gloves during the medication application training:<br>'I am wearing gloves to protect myself from unnecessary exposure to medication.'<br><br>If necessary, don't forget that the patient needs to wash his hands after the training session if having gotten into contact with the medication!<br><br>Explain handling and special aspects of medication.

**Time management of topical therapy**
• Interview the patient about daily activities (job, clothing, etc.)<br>• Decide together which time point during the day fits in the best for topical therapy:<br>- morning/noon/evening<br>• Estimate the time needed every day for the topical therapy. The patient must be aware of time needed (preferably in minutes).

**Onset of therapeutic effect: Expectations of the topical therapy**
• Inform patient about onset of therapeutic effect and expectations.

**Dispense of medication to patient**
• Remind the patient of the dosing regimen.

**Scheduling of next visit**
Schedule next visit; key phrases:<br>a) Lot of information today<br>b) Are there any questions/concerns now about the use of the medication?<br>c) Timeframe for next visit<br>d) For questions/concerns until next visit, patient should feel free to contact you at the telephone number/e-mail address on Patient Card.

Subsequent visits - summary

**Welcome**
Key phrases:<br>• Patient's well-being since last consultation?<br>• Questions/concerns with regard to the application of the medication?<br>• Number of days patient used medication since last visit.<br>• Reassure patient to feel free to contact you at any time also in between consultation visits at the given telephone number/e-mail address that is on his Patient Card.

**Time management of topical therapy**
• Ask patient if there is a need for retraining the application of the topical medication<br>• Ask if time point for treatment chosen during the last consultation is indeed convenient:<br>- morning/noon/evening

**Onset of therapeutic effect: Expectations of the topical therapy**
• Reassure treatment effect onset together and inform patient what to expect further.

**Continued use of medication**
• Explain how the patient should use the medication in the future.

**Dispense of medication to patient**
• Remind the patient of the dosing regimen.

**Scheduling of next visit**
Schedule next visit; key phrases:<br>a) Are there any questions/concerns left unanswered today?<br>b) Timeframe for next visit<br>c) For questions/concerns until next visit, patient should feel free to contact you at the telephone number/e-mail address on Patient Card.
