# Supplementary material for: Development of an adherence-enhancing intervention in topical treatment termed the topical treatment optimization program (TTOP)
Source: Arch Dermatol Res. 2014 Jun 4;306(7):667–76. doi: 10.1007/s00403-014-1475-5 (PMC4139584; doi:10.1007/s00403-014-1475-5)
Supplement: Supplementary file 3 — Supplementary material 3 (PDF 69 kb) [file 403_2014_1475_MOESM3_ESM.pdf]

Development of an adherence enhancing intervention in topical treatment termed the Topical Treatment Optimization Program (TTOP)

Archives of Dermatological Research

Kristian Reich[a], Ulrich Mrowietz[b], Eleni Karakasili[c], Ina Zschocke[c*]

[a]Dermatologikum Hamburg, Stephansplatz 5, 20354, Hamburg, Germany

[b]Psoriasis-Center, Department of Dermatology, University Medical Center Schleswig-Holstein, Schittenhelmstraße. 7, 24105, Kiel, Germany

[c]SCIderm GmbH, Drehbahn 1-3, 20354, Hamburg, Germany

*Corresponding Author:
Ina Zschocke
SCIdem GmbH
Drehbahn 1-3
20354 Hamburg
Germany
Phone: +49-040-55 44 010
Fax: +49-40-55 44 01 291
Email: ina.zschocke@sciderm.com

Online Resource 3 General Patient Encounter Guide

A General Patient Encounter Guide*

Please consider the following when meeting with your patient.

<u>1. Hand washing/other sanitizer</u>

Do it • Make sure your patient sees you do it. • Either one alone is not good enough.

<u>2. Polite greeting</u>

Eye contact and handshake both at start and end of consultation visit. • Acknowledgement of everyone in the room.

<u>3. Sit, lean forward, use silence and be calm</u>

<u>4. Ask open ended questions</u>

For example: ‘How are you today?’ or ‘Is there anything else?’

Don’t interrupt • If there are too many issues, make clear what you have to focus on/what you have time for during this consultation. • However, acknowledge the patient’s needs.

<u>5. When taking the history</u>

Ask how psoriasis affects life and offer sympathy. • If possible, use ungloved hands when assessing affected skin areas.

<u>6) Provide explanations and be open, honest and responding about any aspect of the care you can provide</u>

Tell the patient what and why you are doing. • Tell the patient what comes next •
Inform the patient about the disease • Inform the patient about the therapy • Inform the patient about the effect and side effects that he may expect from the therapy

<u>7) Provide written education materials when/where available</u>

<u>8) Ask if the patient has questions</u> and <u>recognize when a lot of information is being provided</u>

Encourage patient to write down any questions and bring them along to a next visit.

<u>9) Follow up</u>

Provide contact information and opportunity for follow up. • Reinforce the importance of adherence to the provided therapy at every consultation/contact. • Ask patients for feedback.

* This guide has been adapted from E.E. Uhlenhake et al. J. Derm. Treatm. 2010, 21, 6-12.
